# Light and Electron Microscopic Studies on Prenatal Differentiation of Exocrine Pancreas in Buffalo

**DOI:** 10.1155/2016/2414769

**Published:** 2016-02-15

**Authors:** Divya Gupta, Varinder Uppal, Neelam Bansal, Anuradha Gupta

**Affiliations:** Department of Veterinary Anatomy, College of Veterinary Sciences, Guru Angad Dev Veterinary and Animal Sciences University, Ludhiana, Punjab 141004, India

## Abstract

The study was conducted on pancreas of 24 buffalo fetuses collected from abattoir and Veterinary clinics, GADVASU, Ludhiana. The buffalo fetuses were divided into three groups after measuring their CVRL, namely, group I (CVRL between 0 and 20 cm), group II (CVRL above 20 cm and up to 40 cm), and group III (CVRL above 40 cm) and their approximate age was calculated. The tissues were processed for light and ultrastructural studies. In group I, at 1.2 cm CVRL (34 days), the pancreas comprised tubules and solid nest of undifferentiated epithelial cells. At 7.5 cm CVRL (63 days) acinar cells with zymogen granules were observed. These acinar cells varied in shape from columnar to pyramidal. At 12.8 cm CVRL (86 days), parenchyma began to organize into lobes and lobules. The centroacinar cells were observed at 12.8 cm CVRL (86 days). In group II, at 28.3 cm CVRL (137 days), there was extensive branching of tubules that resulted in highly branched ductal tree connecting exocrine secretary units to the duct system. The interlobular and intralobular ducts were well observed at this age yet the intercalated ducts were not completely developed. In group III, exocrine pancreas showed a massive growth at 48 cm CVRL (182 days) with distinct pancreatic lobes and lobules. At 54 cm CVRL (195 days), well developed pancreatic architecture was seen with the presence of extensive development of exocrine part organized in lobes and lobules with interlobular and intralobular ducts whereas the intercalated ducts were observed in 80 cm CVRL (254 days).

## 1. Introduction

Pancreas is a bifunctional organ consisting of an exocrine part organized in acini and a duct system that secretes enzymes for digestion and an endocrine part that secretes hormones like insulin, glucagon, and so forth that helps in glucose homeostasis. Knowledge of development of pancreas is essential to understand congenital pancreatic abnormalities like anomalous pancreatobiliary junction, annular pancreas, and pancreas divisum arising from abnormal histogenesis and morphogenesis occurring during critical period of organization of the organ during prenatal life [1]. Nowadays, the biology of the pancreas has been studied intensely, largely driven by the hope of finding better treatments for devastating pancreatic diseases, such as diabetes mellitus, pancreatitis, and pancreatic adenocarcinoma. In particular, advancements in stem cell technology have recently sparked optimism that diabetes could be cured by harvesting stem cells for therapeutic use. This has led to heightened interest in understanding embryonic development of the pancreas [2].

In literature, prenatal development of exocrine pancreas has been reported in rat [3], pig [4], sheep [5], rabbit [6], and human [7, 8], but very scanty literature is available in buffaloes [9, 10]. So the present research work was conducted. Most of the work on development of exocrine pancreas has been reported in human. Since mammals, birds, reptiles, and amphibians have a pancreas with similar histology and mode of development, the findings of the present research have been discussed with the available literature in human. The recognition of normal development of the pancreas in mammals and other species can help understand congenital anomalies in humans and other species [11]. So, the present research may provide a basic data which can be used to evaluate any abnormality occurring in the development of the pancreas at a critical period of organization.

## 2. Materials and Methods

The present study was conducted on pancreas of 24 buffalo fetuses collected from slaughter house and Veterinary clinics, GADVASU, Ludhiana. The length of the fetuses was measured with the help of inelastic thread as a curved line along the vertebral column between the most anterior part of frontal bone and the rump at ischiatic tuberosity and designated as crown vertebral rump length (CVRL). After measuring the CVRL in centimeters, the approximate age of the fetuses was estimated by using the formula given by Soliman [12] in buffalo. Based on the CVR length, the samples were divided into three groups: group I, CVR length up to 20 cm, group II, CVR length above 20 cm and up to 40 cm, and group III, CVR length above 40 cm. The pancreas was dissected out from the abdominal cavity and the tissues were fixed in 10% neutral buffered formalin and Bouin's fixative immediately after collection. After the fixation, the tissues were processed for paraffin blocks preparation by acetone benzene schedule [13]. The blocks were prepared and the sections of 5-6 *μ*m were cut with rotary microtome. The paraffin sections were stained with hematoxylin and eosin, Masson's trichrome, and Holme's stains.

For electron microscopic studies, fresh tissues were washed and fixed in Karnovsky's fixative. The secondary fixation was done for 2 hours in 2% osmium tetraoxide. The tissues were dehydrated, cleared, infiltrated, embedded, and polymerized. The ultrathin sections (70–90 nm) were cut and stained with uranyl acetate (15 min) followed by lead citrate (10 min) [14]. Finally, the grids with sections were examined under transmission electron microscope.

## 3. Results

**Figure 1:**
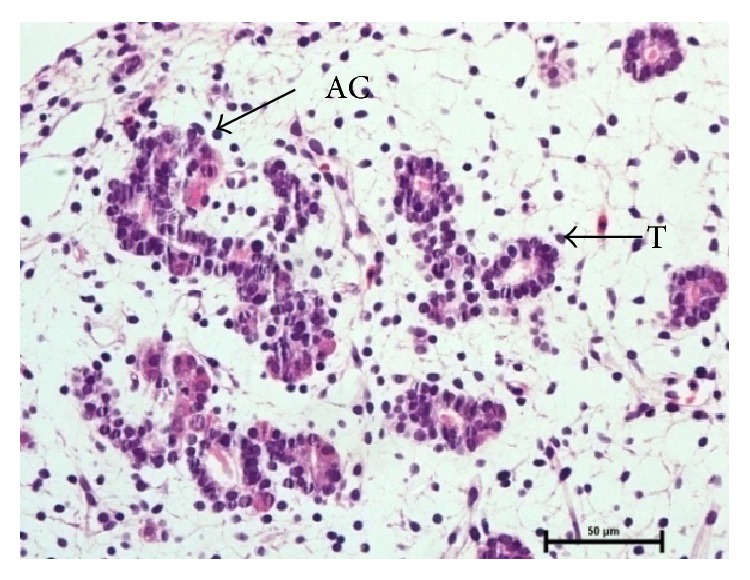
1.2 cm CVRL (34 days) showing primitive tubules (T) and developing acinar cells (AC) in mesenchyme of pancreas. Hematoxylin and eosin ×400.

**Figure 2:**
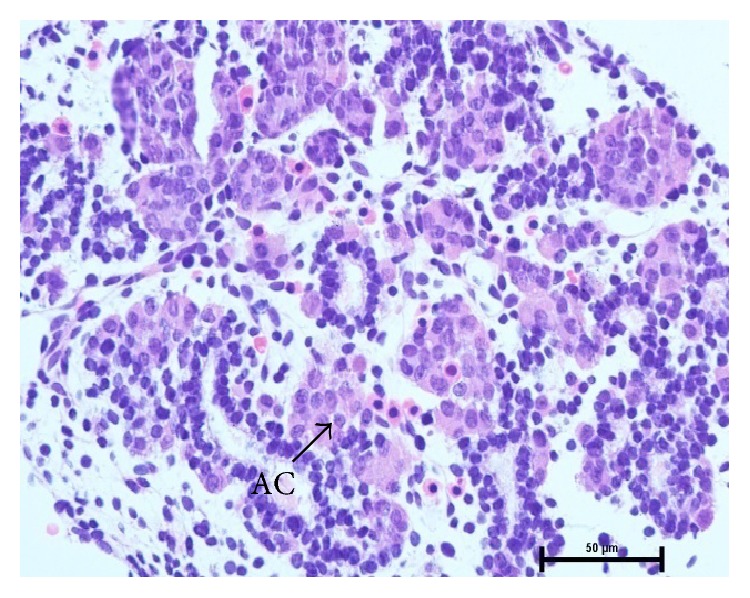
2.6 cm CVRL (40 days) showing development of acinar cells (AC) from primitive tubules. Hematoxylin and eosin ×400.

**Figure 3:**
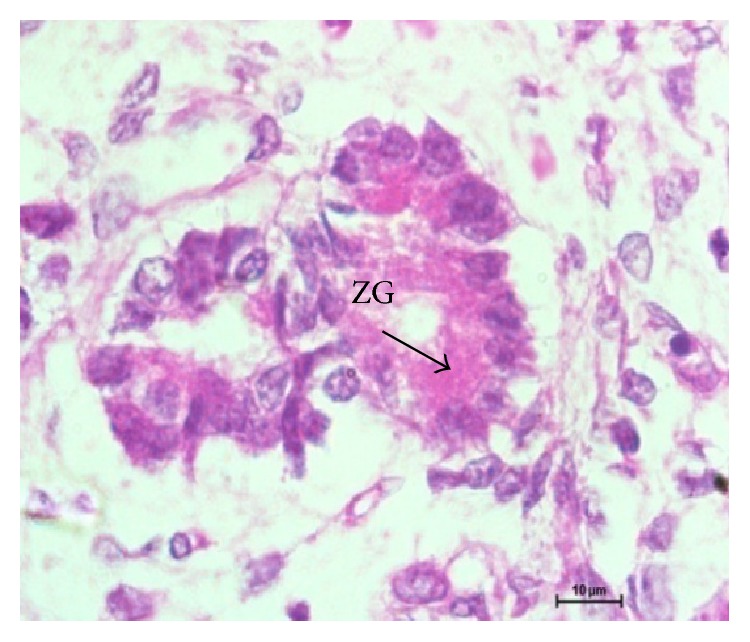
7.5 cm CVRL (62 days) paraffin section showing acini and zymogen granules (ZG) in apical part of cell. Hematoxylin and eosin ×1000.

**Figure 4:**
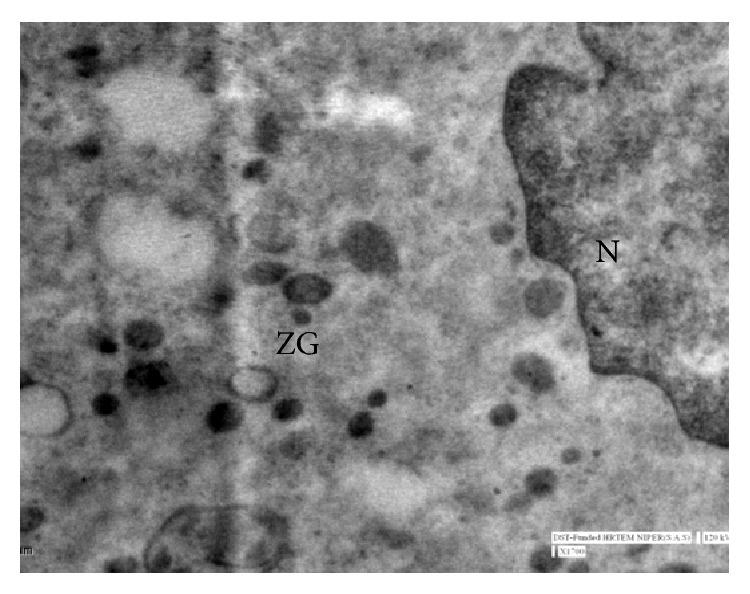
Electron micrograph 7.5 cm CVRL (62 days) showing nucleus (N), developing zymogen granules (ZG), and some of the empty vesicles (EV) in acinar cell ×1700.

In group I, at 1.2 cm CVRL (34 days), the pancreatic parenchyma comprised tubules/ductules and solid nest of undifferentiated epithelial cells in mesenchymal tissue between the developing stomach, duodenum, and mesonephric kidney. The cells of these tubules/ductules were cuboidal in shape having slightly eosinophilic cytoplasm and intensely basophilic nuclei. These tubules/ductules also comprised some cells which were more eosinophilic and these cells represented the differentiating acinar cells (Figure 1) but at this stage no granules were observed in these cells. At 2.6 cm CVRL (40 days), these eosinophilic cells started to assemble in groups but no well defined acini were found (Figure 2). A large number of mitotic figures were observed in these developing cells. The pancreatic duct observed at this stage was lined by simple columnar epithelium. With further branching of pancreatic primitive tubules, the pancreas grew more in size and the luminization of many epithelial buds was observed at 4.2 cm CVRL (48 days). At 7.5 cm CVRL (63 days), there was extensive branching of tubules and these tubules consisted of different types of precursor cells (Figure 3). Newly formed microlumens were initially unconnected but eventually fused into a luminal plexus. Many differentiated acinar cells were grouped to form acini with a well developed lumen. These acinar cells varied in shape from columnar to pyramidal. The supranuclear part of the cell was filled with eosinophilic zymogen granules. The presence of these granules was confirmed by ultrastructural studies and it was found that these granules were of variable sizes and were not fully mature at this stage. Some granules were small, round, and membrane-bound and contained homogeneous electron dense material, whereas others were having moderately electron dense material (Figure 4). Some degranulated vesicles were also present at this stage. Nucleus was large and oval in shape. Endoplasmic reticulum and mitochondria were few in number. The Golgi apparatus was prominent at this stage.

**Figure 5:**
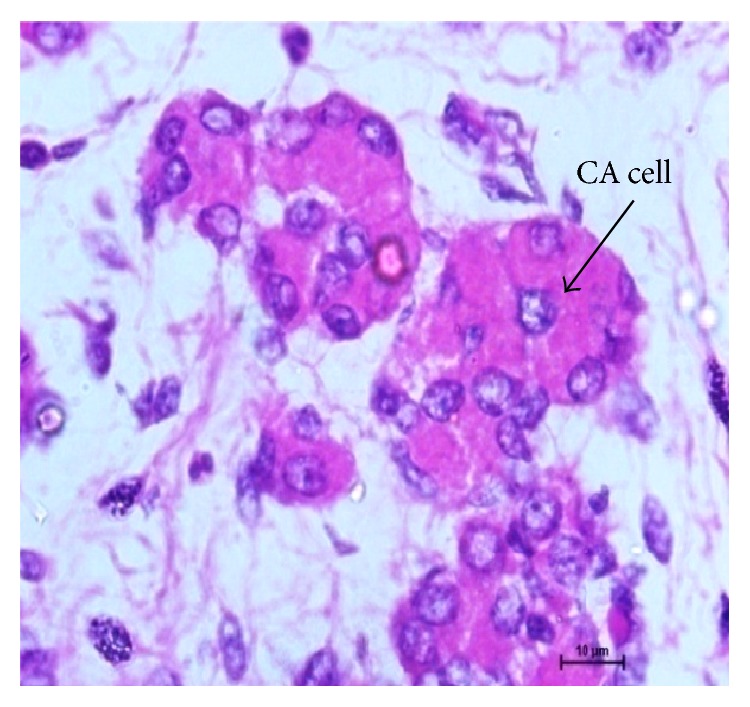
12.8 cm CVRL (86 days) showing centroacinar (CA) cell. Hematoxylin and eosin ×1000.

**Figure 6:**
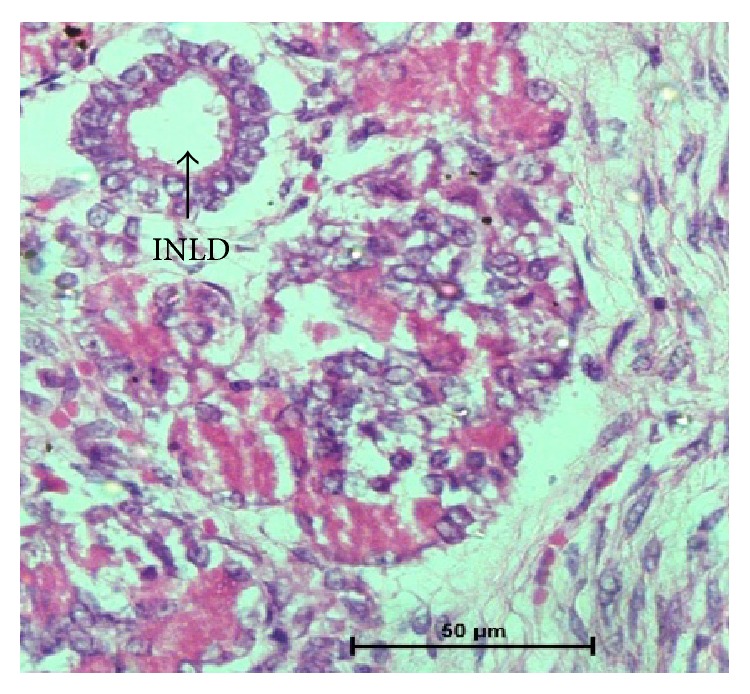
19 cm CVRL (114 days) showing interlobular duct (ILD) and well formed acini (A). Hematoxylin and eosin ×400.

At 9.6 cm CVRL (72 days), there was massive growth of the pancreatic tubules. During the study, the different types of branching patterns were observed. At 10.7 cm CVRL (77 days), the pancreatic duct was lined by simple columnar epithelium and at places glands were observed. At 12.8 cm CVRL (86 days), parenchyma had begun to organize into lobes and lobules with abundant connective tissue (Figure 5). Along with the formation of lobules, interlobular and intralobular ducts were also observed at this age. The intralobular duct was lined by simple cuboidal epithelium, whereas the interlobular duct was having either simple cuboidal or simple columnar epithelium (Figure 6). The cells were tall and pyramidal having a distinct basal lamina. Along with the acinar cells, the centroacinar cells were observed for the first time at this stage. The nuclei of centroacinar cells were basophilic and vesicular. The collagen fibers were better developed.

**Figure 7:**
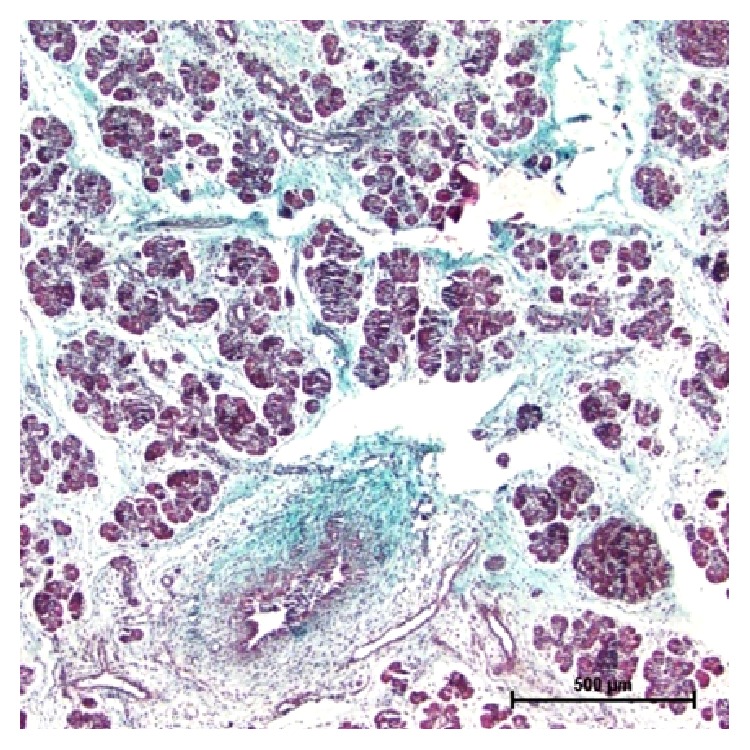
32 cm CVRL (146 days) showing collagen fibers in connective tissue. Masson's trichrome ×40.

**Figure 8:**
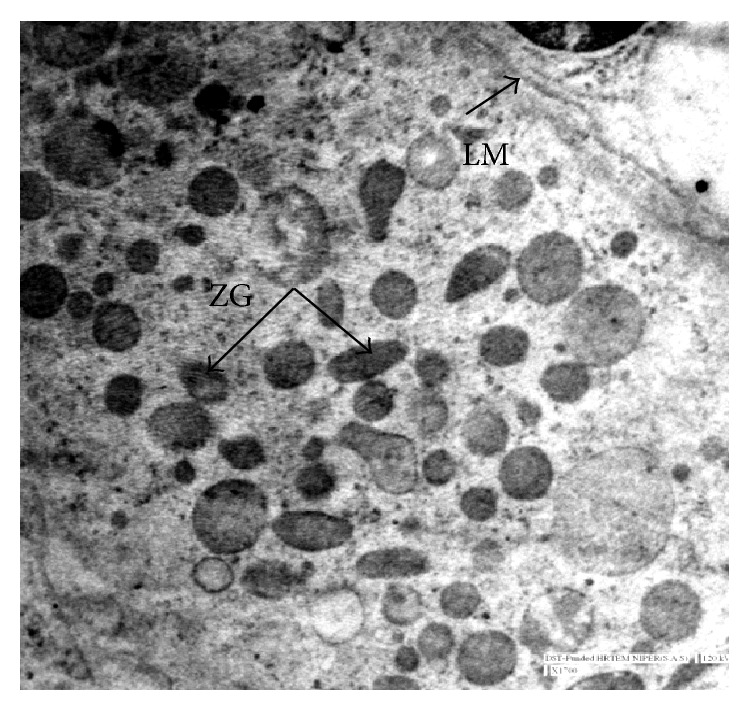
Electron micrograph at 32 cm CVRL (146 days) showing different shapes of zymogen granules (ZG) and lamellar membrane (LM) ×1700.

In group II, at 28.3 cm CVRL (137 days), there was extensive branching of tubules that resulted in highly branched ductal tree connecting exocrine secretary units to the duct system. Although interlobular and intralobular ducts were well developed at this age, the intercalated ducts were not developed as still the acinar cells were budding off from these smaller ducts. At 32 cm CVRL (146 days), acini were more developed and arranged in groups separated by connective tissue (Figure 7). Ultrastructurally, zymogen granules were of different size and shape (Figure 8). These granules were round, oval, spherical, elongated, and spindle-shaped and were located between the Golgi cisternae and the apical membrane. Discharge of granules into the acinar lumen was not observed. The granules were membrane-bound. Some of granules were electron dense and others were moderately electron dense. They contained longitudinal fibrils. In some instances apparent fusion of these elongated granules was observed. Numerous mitochondria and endoplasmic reticulum were present. Moderate amount of glycogen was also observed in acinar cells at this stage especially around the apically located zymogen granules. Nucleus was large and oval in shape. The basolateral membranes were simple with few interdigitations and junctional complexes with neighboring cells. At 35 cm CVRL (153 days), the interlobular duct was lined by simple columnar epithelium with few ciliated cells and goblet cells and intralobular duct was lined by simple cuboidal cells. Ganglia and nerve fibers were also observed in the vicinity of acinar cells.

**Figure 9:**
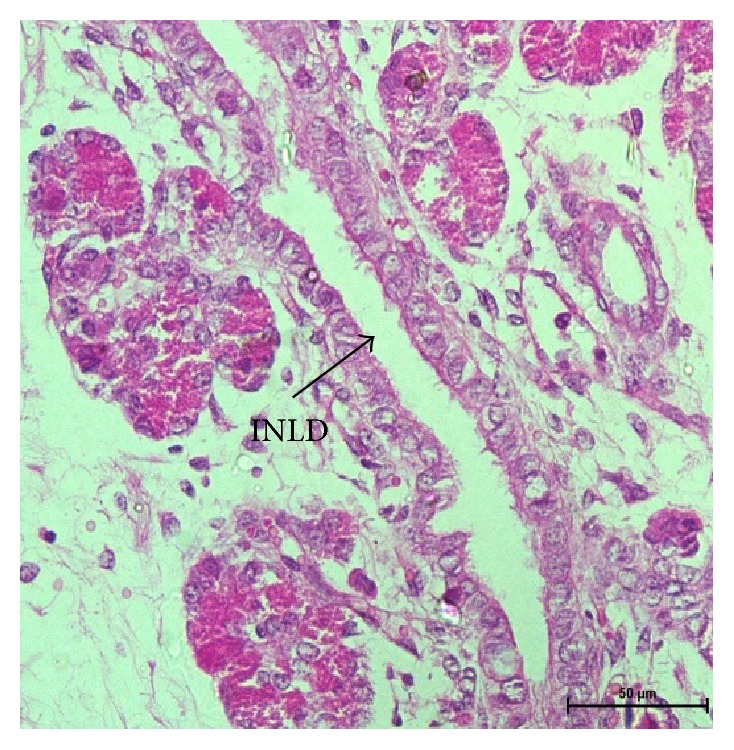
48 cm CVRL (182 days) showing intralobular duct (INLD) and acini (A). Hematoxylin and eosin ×400.

**Figure 10:**
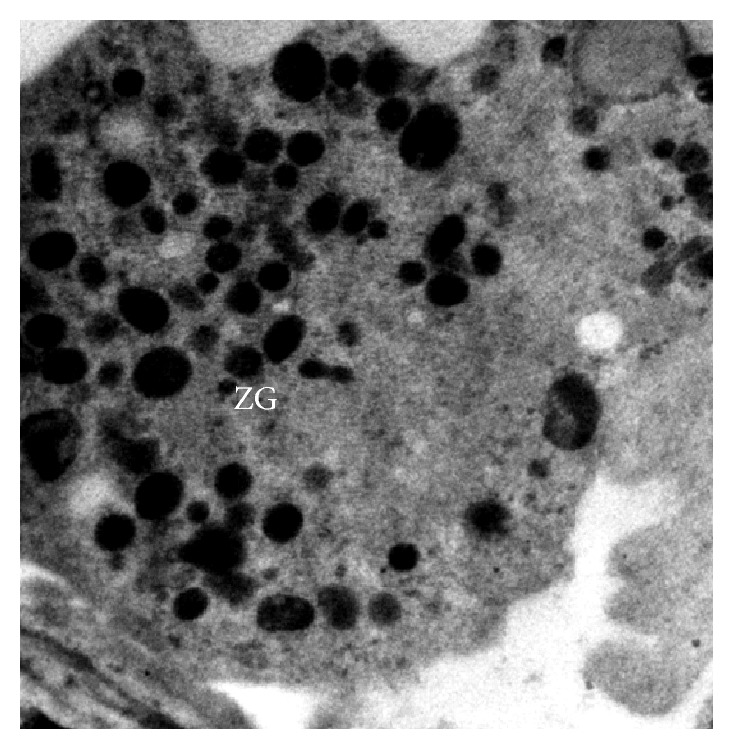
Electron micrograph of foetal pancreas at 48 cm CVRL (182 days) showing electron dense zymogen granules (ZG) in acinar cell ×1700.

**Figure 11:**
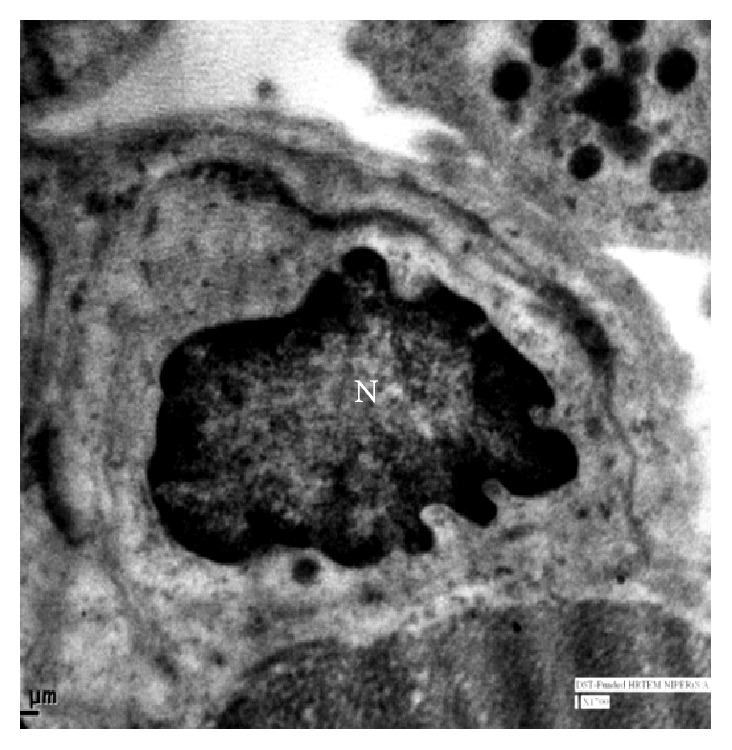
Electron micrograph of 48 cm CVRL (182 days) foetus showing irregular shape nucleus (N) and cell membrane (CM) of centroacinar cell ×1700.

**Figure 12:**
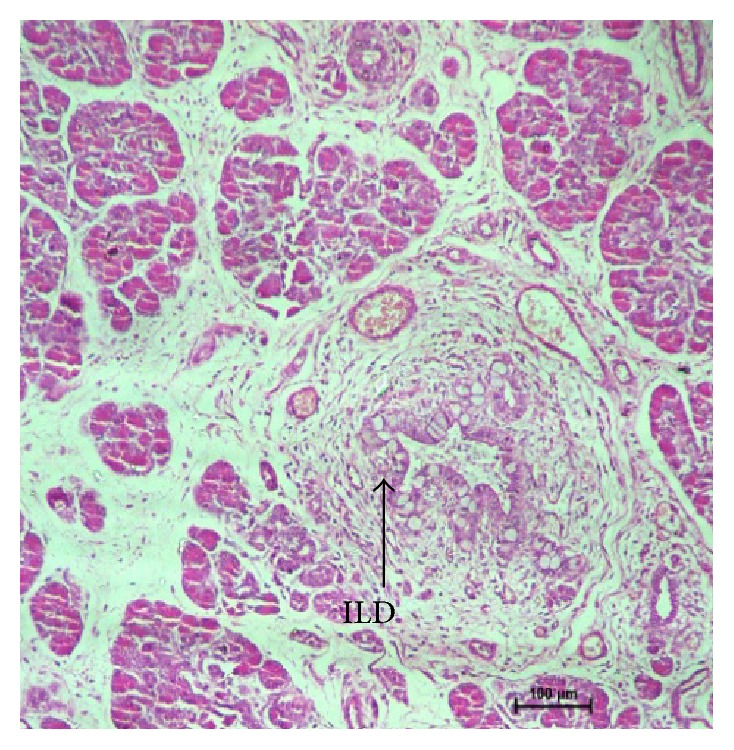
54 cm CVRL (195 days) showing blood vessels (BV) and interlobular duct (ILD) in interlobular stroma. Hematoxylin and eosin ×100.

In group III, exocrine pancreas showed a massive growth at 48 cm CVRL (182 days) (Figure 9). Pancreatic lobes and lobules were very distinct at this stage. Interlobular duct was lined by simple cuboidal epithelium. Their nuclei were large and spherical and with prominent nucleolus. The ducts were still showing the budding of new acinar cells. The nuclei of acinar cells were present near the basement membrane and were having a well developed nucleolus. The upper part of cell was filled with zymogen granules which were more developed than the previous two groups. Ultrastructurally, these granules were large and contained electron dense material (Figure 10) and resembled mature zymogen granules. The granules were almost spherical but they varied in their size. There was a decrease in the number of free ribosomes and a concomitant increase in the amount of rough endoplasmic reticulum. Fewer mitochondria were found in the apex in these mature acinar cells than earlier stages. The junctional complexes were somewhat less prominent. The centroacinar cell had small number of organelles like Golgi apparatus, endoplasmic reticulum, and mitochondria as compared to the acinar cells. The nucleus was irregular in shape (Figure 11). More numbers of blood vessels started to accumulate around the acini and ducts. At 54 cm CVRL (195 days), well developed pancreatic architecture was seen with the presence of extensive development of exocrine part organized in lobes and lobules with interlobular and intralobular ducts (Figure 12). But intercalated ducts were still not observed at this age. The smaller ducts still had the precursors of different cells. A large number of goblet cells were observed in the interlobular ducts. At 70 cm CVRL (232 days), the blood vessels were more developed in the interlobular area with more collagen fibers around them and elastic fibers in the wall of blood vessels. The intercalated ducts were observed in 80 cm CVRL (254 days). These ducts were lined by low cuboidal epithelium. The duct system of the pancreas was well differentiated at this stage. The acinar cells were tall and pyramidal having spherical nucleus with prominent nucleolus present in the basal part of cell.

Micrometrical observations revealed the mean height of acinar cells as 13.4 ± 0.38 *μ*m in group I, 14.03 ± 0.67 *μ*m in group II, and 15.67 ± 0.67 *μ*m in group III, while the mean diameter of acini was 33.5 ± 2.03 *μ*m in group I, 36.4 ± 3.75 *μ*m in group II, and 41.17 ± 6.47 *μ*m in group III. Statistically, the height of acinar cells did not differ significantly between group I and group II and between group II and group III but it differed significantly between group I and group III (*p* < 0.05). So, there was substantial increase in height of acinar cells from group I to group III. The diameter of acini did not differ significantly between group I, group II, and group III (*p* > 0.05).

## 4. Discussion

The solid nest of undifferentiated epithelial cells in pancreatic parenchyma observed at 1.2 cm CVRL (34 days) in the present study has been referred to as cell buds by Conklin [15]. These cell cords are the common precursors of both acinar and islet cells. Such cell cords have been observed by Laitio et al. [16] at 9 weeks and Gupta et al. [17] at 12-13 weeks in human foetal pancreas. Once these cell buds are formed, the next step is the luminization of these epithelial buds. Cleaver and MacDonald [18] have reported that the entire pancreatic tree arose from an endodermally derived protodifferentiated epithelium and multipotent progenitor cells located at branched tips and gave rise to different pancreatic cells including acinar, endocrine, and ductal lineages. Villasenor et al. [19] have demonstrated that the process of pancreatic branching involves the number of cellular events including transient epithelial stratification, dynamic cell polarity shift, asynchronous apical cell constriction, and rosette organization as well as microlumen formation and fusion. Pancreatic tubulogenesis begins with individual cells acquiring apicobasal polarity. Cells develop a defined apical membrane that faces a centre lumen and a basal surface attached to a layer of extracellular matrix. Then these newly formed polarized cells form rosettes wherein adjacent cells are linked by intercellular junctional complexes around a nascent central lumen. Newly formed microlumens are initially unconnected but eventually fuse into a luminal plexus. Remodelling of the luminal plexus produces a tubular network lined by polarized monolayered epithelia attached to basement membrane. It has also been speculated by above authors that mesenchymal tissue is a signaling tissue which helps localize expression of various factors that induce the branching pattern of the pancreatic primitive tubules. Laitio et al. [16] have reported the lumen formation at third foetal month in human pancreas. The presence of zymogen granules in the acinar cells has been reported by Conklin [15] at 110–150 mm CRL in human foetal pancreas and at 12 foetal weeks by Laitio et al. [16]. Tadokoro et al. [1] have reported in rats that the number and density of zymogen granules before birth depend on the serum glucocorticoid concentration. Many enzymes like lipase, trypsin, and amylase are stored in zymogen granules in foetal acinar cells and thus autodigestion of the cells does not occur [3]. It has been well documented that the rough endoplasmic reticulum and the Golgi apparatus are both involved in the early stage of zymogen granule synthesis in the adult [20]. The basolateral membrane junctional complexes have been reported at 20 weeks of gestation in human foetal pancreas [21].

The organization of pancreatic parenchyma into lobes and lobules has been reported by Lucini et al. [9] at 3rd month of gestation in buffalo foetal pancreas. In the present study, the formation of interlobular and intralobular ducts was observed at 12.8 cm CVRL (86 days), whereas in the earlier studies Conklin [15] has reported these ducts at 12.5–14.5 weeks and Gupta et al. [17] have reported them at 18 weeks of age in human foetal pancreas. The pancreatic duct has been observed at 43 days of gestation in human foetal pancreas [22]. With further advancement, the size and shape of zymogen granules were changed. Laitio et al. [16] have described different shapes of granules in human foetal pancreas at 12–16 weeks. Structurally the zymogen granules were fully formed so it may be suggested that these granules may secrete enzymes in the new born calves that may help in digestion of milk in new born animals. Presence of neuronal elements, that is, nerve fibres and ganglion, helps in pancreatic exocrine secretion through parasympathetic stimulation [23]. There was massive growth of exocrine pancreas at 48 cm CVRL (182 days) with well developed acini having better developed zymogen granules as reported earlier by Inagaki et al. [3] in foetal and neonatal rat pancreas and by Laitio et al. [16] in human foetal pancreas. In the present study well developed duct system was observed at 80 cm CVRL (254 days), whereas in an earlier report by Singh and Sethi [10] well established duct system has been reported at 75 cm CVRL (243 days) in buffalo foetal pancreas.

Micrometrical values of diameter of acini observed in present study in all the groups are higher than those reported by Prashar [24] who has reported mean diameter of acini as 22.37 ± 0.74 *μ*m in neonatal buffalo pancreas. So it may be inferred that diameter of acini is increased with advancement of gestation period but after the birth it decreases.
